# Combined extended reality and reinforcement learning to promote healthcare and reduce social anxiety in fragile X syndrome: a new assessment tool and a rehabilitative strategy

**DOI:** 10.3389/fpsyg.2023.1273117

**Published:** 2023-12-20

**Authors:** Fabrizio Stasolla, Anna Passaro, Mariacarla Di Gioia, Enza Curcio, Antonio Zullo

**Affiliations:** ^1^University “Giustino Fortunato” of Benevento, Benevento, Italy; ^2^Universitas Mercatorum of Rome, Rome, Italy

**Keywords:** fragile X syndrome, healthcare, artificial intelligence, technologies, quality of life, reinforcement learning (IRL)

## Introduction

Fragile X syndrome (FXS) is a rare genetic disease caused by mutations in the fifth untranslated region of the FMRI gene situated on the Xq27.3 site, resulting in an expansion of cytosine–guanine–guanine (CGG) trinucleotide repeats. Typically, the CGG segment is repeated between 5 and 40 times in the normally developed population. Conversely, it is usually repeated over 200 times in FXS (i.e., full mutation). Individuals whose CGG repletion is comprised between 55 and 200 times present the premutation (Symons et al., 2003; Crawford et al., 2020; Marschik et al., 2022). The disease affects ~1 in 2,500 male individuals and 1 in 4,000–6,000 female individuals (Oliver et al., 2017). The excessive CGG repeats cause the FMR1 gene to be methylated, resulting in reduced production of the protein FMRP. Because the FXS is an X-linked neurodevelopmental disorder, it is more likely to be observed in male individuals than in female individuals (Adams and Oliver, 2011; Alusi et al., 2022). It represents the most known inherited cause of intellectual disabilities (IDs). Approximately 60% of FXS individuals present an autism spectrum disorder (ASD) comorbidity, while attention deficit hyperactivity (ADHD) is commonly observed in 70% of people with FXS (Kenny et al., 2022; Shaffer et al., 2023). The phenotype is characterized by an elongated face, a high-arched palate, large ears, muscular hypotonia, connective tissue dysplasia, mitral valve prolapse, and joint hypermobility (Cregenzán-Royo et al., 2022). Beyond cognitive impairments, language delays are commonly acknowledged. Adaptive skills are negatively affected, and social abnormalities are frequently observed (Van der Lei and Kooy, 2022). Behavioral difficulties, including poor eye contact, self-injury, aggression, and stereotypic, repetitive, and pervasive behaviors, are additionally reported (Marlborough et al., 2021; Niescier and Lin, 2021). Anxiety is also documented in FXS, with over 80% of the male subjects meeting the criteria for one anxiety disorder and over 60% of male subjects meeting the criteria for multiple anxiety disorders (Alusi et al., 2022; Chen Y. S. et al., 2022). The most frequent categories of anxiety disorders detectable in FXS are selective mutism and specific and/or social phobia. Approximately 60% of the male subjects with FXS show clinically relevant traits of social anxiety (Aishworiya et al., 2022; Chen C. C. et al., 2022).

Anxiety disorders are commonly recognized, with an approximate prevalence of 12 months in 18% of the general population. Children, adolescents, and adults with ID are at least four times more likely to meet the criteria for anxiety than those without ID (Tolmacheva et al., 2022). People with genetic diseases face a higher risk (Kat et al., 2022). Social anxiety is defined as a specific anxiety disorder that evidences persistent features of fear in social contexts (Kat et al., 2022). Exposure to unfamiliar people or environmental conditions is additionally included (Edwards et al., 2022). Eventual scrutiny by others and avoidance of social circumstances are embedded (Protic et al., 2022). Social anxiety represents one of the most common anxiety disorders, with a 12-month prevalence of 7% in the general population and a highly increased rate in people with rare genetic diseases. For instance, in individuals with FXS, as reported above, ~60% meet the diagnostic criteria of social anxiety. The rate is 35 times higher if compared with other idiopathic IDs (DaWalt et al., 2022).

Crawford (2023) suggested a conceptual model of social anxiety in FXS, including five constructs associated with it, namely, (a) physiological arousal, (b) sensory sensitivity, (c) emotion dysregulation, (d) cognitive flexibility, and (e) intolerance of uncertainty. The model emphasizes a multilevel pathway to social anxiety based on a biologically aroused state that, combined with sensory sensitivity, predisposes people with FXS to an inability to manage negative emotions. Consequently, the acceleration of physical processes compromises the capacity to regulate the deceleration of emotional responses. Built on this state of arousal and emotion, the model conceptualizes that individuals with FXS are unable to shift attention away from threatening stimuli, and an intolerance for the uncertainty of everyday life events or circumstances considered threatening is evidenced. A hierarchical element was postulated. Thus, emotional dysregulation is downstream of physiological excessive arousal, while sensory sensitivity, cognitive inflexibility, and intolerance to uncertainty are downstream of emotion dysregulation. This situation may be viewed as deleterious for the individuals' quality of life, with an increased burden on parents and caregivers, and their healthcare might be significantly hampered (Gabis et al., 2023; Klusek et al., 2023; Prior et al., 2023; Wall et al., 2023). To tackle this issue, one may envisage technology-aided interventions and/or artificial intelligence-based setups (Stasolla et al., 2017; Movaghar et al., 2022).

Within new technologies, extended reality (XR) includes computer-generated environments merging the physical and virtual worlds and/or creating an entirely virtual experience for users. XR encompasses the intersection of three different technologies, such as augmented reality (AR), virtual reality (VR), and mixed reality (MR). By merging the aforementioned technologies, XR provides users with a broad range of immersive opportunities across real- and virtual-based environments (Ren et al., 2023). Among AI-based programs, reinforcement learning (RL) as part of machine learning conceives an artificially intelligent agent capable of constantly interacting with the user and being reinforced by him/her; it is capable of constructive learning and can continuously calibrate the difficulty of the task for the user (Nissan et al., 2023). Although highly promising and constituting an invaluable educational and rehabilitative resource, the use of such technologies in neurodevelopmental disorders or developmental disabilities is quite sparse (Pires et al., 2022). Specifically, by inserting FXS, XR, and RL as keywords in Scopus and even merging their combinations, no records were found.

In line with the above, the objective of this opinion paper was to propose a new diagnostic tool and a plausible rehabilitative strategy to promote the healthcare of individuals with FXS and reduce the burden on parents and caregivers. In addition to an assessment tool to differentiate emotion regulation and tolerance to social anxiety, we suggest a combined strategy between XR and RL to promote constructive engagement in individuals with FXS (Stasolla et al., 2014a,b). In the following sections, we first describe and detail the functioning of XR and RL; subsequently, we argue for the combination of both as an assessment tool and a rehabilitative approach. The implications were critically discussed.

## Extended reality

A wide range of terms are commonly used to summarize different virtual reality and related technologies that represent simulated real worlds built through computers, mobile devices, and wearable devices (Chen and Geschwind, 2022; Tan et al., 2022). XR is an umbrella term that encompasses all categories of real and virtual environments. Thus, AR, MR, and VR represent different modalities with different features. However, a considerable level of overlap may be found in the literature. Accordingly, non-experts may easily confound and use interchangeably one with the other (Park et al., 2022). For practical reasons, we avoid explaining each specific technology. Rather, by examining the existing literature, we found six records including XR and developmental disabilities (Camfield et al., 2016; Evans, 2018; Ropar et al., 2018; Stevens, 2018; AlMusawi et al., 2019; Park et al., 2022), and only two records using XR and neurodevelopmental disorders (Atkinson et al., 2003; Kim and Koh, 2016). Nevertheless, those technologies were largely and successfully adopted for developmental disabilities and neurodevelopmental disorders (Atkinson et al., 2003; Kim and Koh, 2016; MintzHemed and Melosh, 2023; Wu et al., 2023). Their use may be considered a crucial and invaluable resource because those technologies provide high levels of ecological validity (i.e., with situations similar to real life) and provide researchers and clinicians with practical knowledge through behavioral tracking and experimental control (Liu et al., 2022; Perera et al., 2022). Matched with serious games (SRs), those technologies may easily pursue educational, funny, diagnostic, and rehabilitative purposes (Hossain et al., 2016; Ávila et al., 2017; Maio et al., 2022a). By merging FXS and XR in Scopus, no records were found, suggesting that further research in this specific area was warranted. For example, one may argue that the healthcare and quality of life of individuals with FXS may be relevantly improved and the burden of parents and caregivers similarly reduced (Celesti et al., 2022; Maio et al., 2022b).

## Reinforcement learning

Constantly interacting with the participant and calibrating the difficulty of a proposed activity or task to the individual's skills and performances, as part of machine learning, may be considered crucial for the success of an assessment or a rehabilitative strategy. Currently, computerized systems frequently include specific processes with progressive difficulties throughout sessions. Usually, those difficulties increase step by step according to predefined rules evaluated by a neuropsychologist who assesses the participants' performance. For example, such computerized systems daily adapt to the activities and increase the difficulty whenever a predefined threshold is reached. However, the order in which the specific parameters of the task are modified is fixed. Therefore, the characteristics of the unique participant are almost ignored. Thus, the peculiarities of the participants are neglected (Zini et al., 2022).

Broadly speaking, the basic concept is that in computerized systems, an RL AI agent is linked to a unique participant for each suggested activity or task. Thus, the agent continuously interacting with the participant while the exercise is running will be able to provide a rigorously tailored and customized difficulty, which is optimal for the unique participant. The agent is capable of learning from the specific performance of the participant through algorithms and parameters that are continuously adapted to the performance. A customized policy for each participant will be targeted, considering the ongoing performance. Specifically, the individuals' performance while completing the task plays the role of highly positive reinforcement useful to progressively enhance a policy by modifying the values of the parameters over time. Accordingly, the difficulty of the activity or task is optimized. That is, the task can be adapted individually, and the policy adopted to vary the parameters and to optimize the intervention can be independently learned for each participant (Coronato et al., 2020).

In light of the above, one may consider the combined integration between XR and RL. It may be conceived as an invaluable technological resource because the artificially intelligent agent may personalize the difficulty of the activities and/or tasks for the user, while the XR setups may enable an immersive environment similar to real life. The integration and combination of different technologies have been recently postulated and suggested by Stasolla et al. (2022, 2023a,b). Next to the positive outcomes for the participant, one may reasonably argue on the reduction of both caregivers' and families' burdens (Lopez-Vargas et al., 2019; Stasolla et al., 2021; Teriö et al., 2022). The innovative characteristic may be emphasized by the combined integration of both technologies, whose application and implementation may be adopted for both assessment and rehabilitative objectives in the early stages of social anxiety among FXS individuals. Furthermore, the emotional and cognitive competencies of individuals with FXS might be accurately investigated (Raspa et al., 2017; Bartholomay et al., 2019).

## How should it work

The convergence of extended reality (XR) and reinforcement learning (RL) for the betterment of health and reduction of social anxiety in individuals with fragile X syndrome necessitates a multifaceted approach that integrates cutting-edge technologies and a profound understanding of the syndrome itself (Pons et al., 2022).

Initiating the process with an exhaustive exploration of fragile X syndrome lays the groundwork for an enhanced comprehension of its symptoms, risk factors, and the intricate challenges faced by those affected. Following this, the development of a three-dimensional virtual reality (VR) or mixed reality (MR) environment comes into play, aimed at replicating authentic social scenarios. These scenarios could encompass interactions with others, work-related situations, or even gaming scenarios. Integration of sensors within the virtual environment is then employed to capture biometric data, including heart rate, perspiration, and facial expressions during individuals' engagements within the virtual realm. The implementation of RL agents follows suit, designed to perpetually monitor the amassed biometric data. These agents discern instances when an individual with fragile X syndrome exhibits indications of social anxiety (Dechant et al., 2017).

Upon the detection of social anxiety, RL agents step in with personalized interventions. This may involve offering tailored suggestions, facilitating relaxation exercises, or adjusting the intensity of social interactions within the virtual environment to align with the individual's specific needs. A pivotal aspect involves continuous learning by RL agents, allowing them to adapt and refine their responses based on the outcomes of interactions over time. Therapeutic involvement is also woven into the fabric of this approach, with mental health professionals or therapists seamlessly integrated into the virtual environment. Their role is to provide direct support and counseling as necessary. The process includes a robust system for the evaluation and improvement of individuals' progress, utilizing biometric sensor feedback to collect data on mental health advancements over time (Yao et al., 2020).

An emphasis on ethics and privacy underscores the importance of stringent privacy policies and ethical considerations, empowering individuals to control their data and participate in the virtual environment. To ensure a comprehensive and ethical framework, interdisciplinary collaboration is pivotal. Experts in medicine, psychology, artificial intelligence, ethics, and XR development collectively contribute to shaping this holistic approach. In essence, the overarching goal of merging XR and RL is to forge a secure and personalized environment for individuals grappling with fragile X syndrome. The ultimate aim is to champion mental health and alleviate social anxiety. However, it is crucial to underscore that this innovative approach necessitates further research and technological advancements to transition from concept to practical and effective reality (Warin, 2022).

Considering the multilevel pathway model suggested by Crawford (2023) above, an optimal starting point for an artificial agent working according to RL rules usually comes from a fully randomized policy criterion. That is, an initial identical probability is assigned to all considered actions that the agent can carry out. The parameters are identified by the five basic elements acknowledged by the model. An identical initial probability is randomly assigned to each parameter/element of the model. Two different outcomes are sought: (a) a successful consequence is empirically recorded because an optimal policy has been designed with a long interaction between the participant and the system or (b) a failure is detected because an initial poor effect of the learning process is planned and frustration by the participant is empirically documented (Ciampi et al., 2022). To overcome this issue, one may envisage an association between category policies and exercises or tasks. The category policy should include a probability of actions that is not the ideal one but very close to it if compared to the fully randomized policy. Starting from the task category policy, an individualized participant–agent interaction would be fostered, and the accuracy of the ideal personalized policies can be programmed (Stasolla and Di Gioia, 2023).

XR-based setups may enable, on the other hand, an individual with FXS to have a fully immersive experience similar to real life. For instance, different experimental conditions eliciting different emotions can be built. Based on the participants' preferences (Lancioni et al., 2022), one may consider different scenarios rigorously adapted to the individual level of social functioning (i.e., including cognitive, emotional, and motor functioning). For FXS individuals with severe to profound intellectual disabilities, one may design a basic recognition of facial expressions, representing different emotions. The participant would be required to correctly identify the solicited emotion in each trial. For individuals with moderate intellectual disabilities, one may consider request and choice access to preferred items eliciting different emotions. A hierarchical system requesting different choices to be performed by the participant would be proposed (Lancioni et al., 2008b, 2022). For individuals with mild cognitive impairment, one may propose cognitive tasks or exercises mediated and supported by customized technology (Hocking et al., 2023).

**Figure 1:**
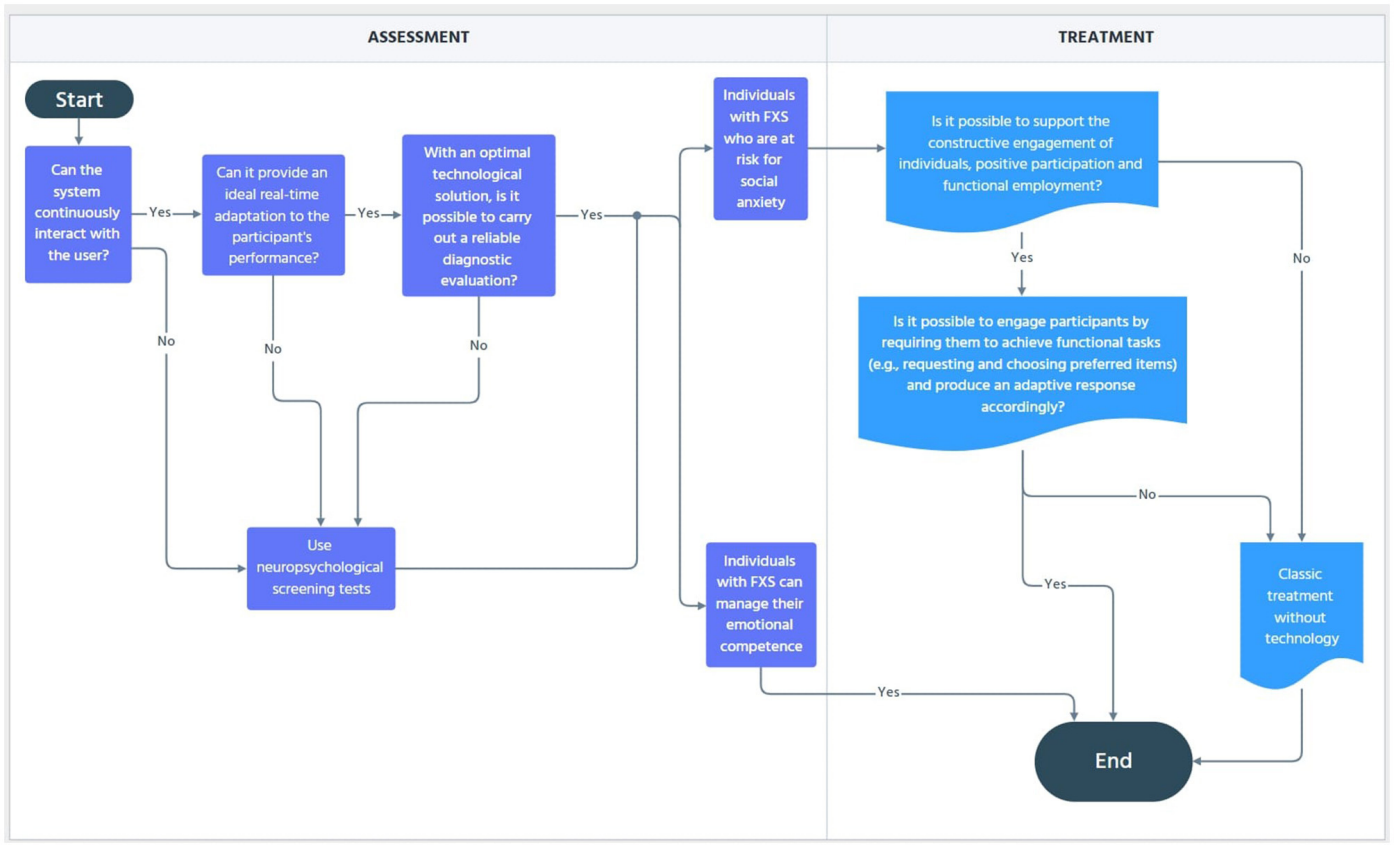
Flow chart diagram of the process for reaching the proposed assessment and recovery objectives.

The opportunities detailed above may be helpful to easily pursue a dual goal. An assessment of individuals who are at risk of social anxiety may be reached. A rehabilitative strategy can be adopted to promote constructive engagement, positive participation, and functional occupation (Stasolla et al., 2014a). Different solutions tailored to the participants functioning and preferences might be encouraged (Caffò et al., 2016). In summary, in a unique session of intervention, a combined solution of XR and RL would provide the participant with an optimal technological solution depending on the global functioning of the participant, and at the same time, the artificially intelligent agent would constantly calibrate the difficulty of the task. With the illustrative examples detailed above, one can differentiate between an individual who is capable of managing his/her emotional states and an individual who is at risk of social anxiety (i.e., social value). Once differentiated, one might propose a rehabilitative intervention with further experimental conditions (i.e., see above) to promote adaptive responding and constructive engagement in individuals with FXS (Caffò et al., 2016; Raspa et al., 2020; Ellis et al., 2021). To enhance the reader's ability to follow the logic with each possible outcome, we inserted a graphical representation of the proposed functioning in Figure 1.

## Discussion

We proposed a combined technology integrating XR and RL in a unique intervention with a dual purpose: (a) the assessment and (b) the rehabilitation of cognitive and emotional competencies in individuals with FXS. Regarding social anxiety, we described the model suggested by Crawford (2023). Moreover, we argued about the combined mechanism aimed at identifying cognitive and emotional skills in individuals with FXS. Finally, we detailed a new diagnostic tool and a new rehabilitative strategy for assessment and recovery objectives. We provided some illustrative examples to identify people with FXS who are capable of independently managing their emotions and individuals with FXS who are at risk of developing social anxiety. Furthermore, depending on the individual's functioning, we presented different possible scenarios and/or experimental conditions. The proposal may suggest the following considerations.

First, a new assessment tool was tentatively structured. The system may be considered valuable because by continuously interacting with the user, it can provide an ideal adaptation in real-time to the performance of the participant. With an optimal technological solution, a reliable diagnostic evaluation could be made. One may argue that an early assessment, eventually supported by neuropsychological screening, can be carried out. It could be viewed as critical to differentiate between individuals with FXS who could manage their emotional competence mediated by coping strategies and individuals with FXS who were at risk of social anxiety (Lancioni et al., 2009a,b, 2010; Tarver et al., 2021; Crawford et al., 2023; Jones et al., 2023).

Second, based on a rehabilitative point of view, one may support the individual's constructive engagement, positive participation, and functional occupation. Thus, by involving the participants in social and/or emotional conditions as described above, people with FXS and ID may be requested to achieve functional tasks (e.g., request and choice of preferred items) and produce an adaptive response accordingly (Lancioni et al., 2009a, 2010).

Third, the participants' quality of life may be significantly improved, and families' or caregivers' burdens may be similarly reduced. One may argue that the healthcare of individuals with FXS can be favorably supported. However, the integration in clinical settings was recently conceptualized (Jones et al., 2023) should be carefully evaluated. Furthermore, its suitability in daily contexts (e.g., home and educational environments) should be additionally considered.

Fourth, clinical and social validity should also be included. For example, social validation procedures involving external experts such as neurologists, psychologists, parents of children with developmental disabilities, physiotherapists, and/or speech/occupational therapists are warranted. External validity, including systematic comparisons with idiopathic disabilities or other developmental disabilities, should be viewed as mandatory (Lancioni et al., 2004, 2007, 2008a, 2009a; Stasolla et al., 2014a).

## Limitations and future research

Despite the beneficial perspectives, caution is highly recommended. Thus, different limitations can be recognized. First, empirical data in our proposed combination between XR and RL are currently missing. Systematic investigations of FXS individuals are mandatory. Either group comparisons or longitudinal single-subject-based studies, as well as several clinical trials, should be conducted. Second, both assessment and rehabilitative goals should be addressed daily. Third, the acceptability of young and older adults with FXS should be considered. Fourth, suitability and sustainability in daily settings should be a research priority. Fifth, affordability in daily contexts should be adequately evaluated. Sixth, the proposed tool should be carefully assessed through a large sample of participants, including other idiopathic ID and/or different genetic diseases (e.g., Angelman, Cornelia de those with Lange, and Rett syndromes). Seventh, it should be noted that FXS is also associated with hypersensitivity to sensory stimuli, and this could impose some challenges while also providing opportunities by altering the amount of XR sensory stimulation. To overcome this issue, one may argue that the combined RL and VR may provide individuals with FXS in a highly continuous and rigorously customized and tailored fashion. Thus, an adapted and updated version of Crawford's Model (Crawford, 2023) may be envisaged. A sixth parameter, including a further detailed customized sensitivity, may be embedded. The RL-based agent would calibrate the individual's sensitivity to such a parameter. An optimal VR setup would be adopted. Further evaluations might be assessed with neuropsychological and neurophysiological measures (e.g., event-related potentials, neural oscillations, and animal models). Outcomes of clinical trials, profiles, and developmental trajectories may additionally indicate the degree of sensitivity (Budimirovic et al., 2017; Sinclair et al., 2017; Jenner et al., 2023).

In light of the above, future research perspectives should include the following topics: (a) to empirically investigate the use of a combined XR and RL tool to evaluate emotional competence and social anxiety in FXS, (b) to assess the suitability and sustainability on environmental, financial, and human resources, (c) to consider affordability through social validation procedures, including psychologists, neurologists, clinicians, researchers, and caregivers as external and expert raters, and (d) gradually carry out systematic reviews and meta-analysis within this specific framework.

## Conclusion

A combined integration between XR and RL was suggested to pursue the dual objective of evaluation and rehabilitation in FXS individuals. An early assessment with the combined technology may be viewed as critical to preventing social anxiety and emotional dysregulation. Additionally, the combined technology can be used as a useful rehabilitative strategy, providing a continuous and rigorously tailored adaptation to the activity's complexity. A systematic matching between different technologies (e.g., assistive technology, telerehabilitation, and VR) has been recently proposed (García et al., 2021).

An adequate evaluation of the personalized options for each use is warranted. A remote supervised control may be implemented (Bernini et al., 2023a). Specific adaptations, suitability investigations, and satisfaction experiences should be relevantly issued within this framework (Bernini et al., 2023b).

## Author contributions

FS: Conceptualization, Writing – original draft. AP: Writing – review & editing. MD: Supervision, Writing – review & editing. EC: Supervision, Writing – review & editing. AZ: Supervision, Writing – review & editing.
